# Probiotics in Irritable Bowel Syndrome: An Up-to-Date Systematic Review

**DOI:** 10.3390/nu11092048

**Published:** 2019-09-02

**Authors:** Hanna Fjeldheim Dale, Stella Hellgren Rasmussen, Özgün Ömer Asiller, Gülen Arslan Lied

**Affiliations:** 1 Centre for Nutrition, Department of Clinical Medicine, University of Bergen, 5009 Bergen, Norway; 2 Division of Gastroenterology, Department of Medicine, Haukeland University Hospital, 5021 Bergen, Norway; 3 National Centre of Functional Gastrointestinal Disorders, Haukeland University Hospital, 5021 Bergen, Norway; 4 Department of Gastroenterology, Ankara University Faculty of Medicine, Mamak Ankara 06620, Turkey

**Keywords:** irritable bowel syndrome, probiotics, gut microbiota

## Abstract

Irritable bowel syndrome (IBS) is a frequent functional gastrointestinal disorder, and alterations in the gut microbiota composition contributes to symptom generation. The exact mechanisms of probiotics in the human body are not fully understood, but probiotic supplements are thought to improve IBS symptoms through manipulation of the gut microbiota. The aim of this systematic review was to assess the latest randomized controlled trials (RCTs) evaluating the effect of probiotic supplementation on symptoms in IBS patients. A literature search was conducted in Medline (PubMed) until March 2019. RCTs published within the last five years evaluating effects of probiotic supplements on IBS symptoms were eligible. The search identified in total 35 studies, of which 11 met the inclusion criteria and were included in the systematic review. Seven studies (63.6%) reported that supplementation with probiotics in IBS patients significantly improved symptoms compared to placebo, whereas the remaining four studies (36.4%) did not report any significant improvement in symptoms after probiotic supplementation. Of note, three studies evaluated the effect of a mono-strain supplement, whereas the remaining eight trials used a multi-strain probiotic. Overall, the beneficial effects were more distinct in the trials using multi-strain supplements with an intervention of 8 weeks or more, suggesting that multi-strain probiotics supplemented over a period of time have the potential to improve IBS symptoms.

## 1. Introduction

Irritable bowel syndrome (IBS) is a frequent functional gastrointestinal disorder (FGID), calculated to affect around 11.2% of the world’s population [1]. Symptoms include bloating, flatulence, abdominal pain, or discomfort associated with a change in bowel habits (diarrhea, constipation, or mix). The pathophysiology of IBS is not clearly understood, and it is suggested that the condition is multifactorial, affected by environmental, inherited, and psychosocial factors. Suggested mechanisms include visceral hypersensitivity, dysfunction in the gut–brain axis, disturbances in the epithelial barrier integrity causing abnormal change in intestinal permeability, altered gastrointestinal motility, immune activation, abnormal enteroendocrine signaling, as well as dysbiosis in the gut microbiota [2,3]. The diagnosis of IBS is based on exclusion of other severe gastrointestinal disorders and fulfilling of the Rome criteria, a collection of symptom-based diagnostic criteria for IBS and other FGIDs [4].

Medical treatment of IBS is most commonly based on targeting the predominant symptom experienced by the patient. In addition, a diet low in fermentable oligosaccharides, disaccharides, monosaccharides, and polyols (FODMAPs) has been shown to improve IBS symptoms and is currently a recommended dietary strategy [5]. Although IBS is a non-fatal condition, the symptoms are experienced as troublesome for those affected and the condition is associated with increased rates of depression and anxiety, as well as economic challenges, hence often leads to severe reduction in quality of life (QoL) [6]. Neither pharmacological treatment nor diet changes tend to completely eliminate symptoms, therefore alternative approaches to improve symptoms and better life for those affected are of great need [7].

Alteration in the gut microbiome is suggested as a likely contributor to IBS, a concept arisen from clinical observations of symptoms developing after an infection, commonly described as post-infectious IBS [8,9]. Small intestinal bacterial overgrowth often causes symptoms similar to those of IBS, in particular bloating in relation to food intake [10]. Studies comparing the gut microbiota of IBS patients to healthy controls have suggested an altered microbiota profile in IBS [11,12,13,14], and specific gut microbiota profiles have been associated with particular symptoms and severity of disease [15,16].

Probiotics are defined as “live microorganisms that, when administered in adequate amounts, confer a health benefit on the host” [17]. The concept of probiotics was, for the first time, suggested in 1908 by Elie Metchinkoff, a Russian Noble Laureate who observed that consumption of fermented foods containing lactic acid bacteria had a beneficial effect on human health. Based on his own theory that lactic acid could prolong life, he consumed sour milk every day, and was the one to first name it yoghurt [18]. Since then, the effects of probiotics have been widely investigated in a broad spectrum of diseases and are currently suggested as a possible treatment or prevention in several gastrointestinal disorders [19,20].

The exact mechanisms of probiotics in the human body are currently only partly known. Probiotics have been suggested to act through inhibition of pathogenic bacteria overgrowth and prevention of pathogenic invasion of the host, improvement of gut barrier function and receptor interactions, as well as production or secretion of substances such as short chain fatty acids (SCFAs) and neurotransmitters [21]. Studies in both animals and humans have suggested that different strains of probiotics may improve abdominal pain and reduce visceral hypersensitivity by modulation of expression of neurotransmitters and receptors involved in the modulation of pain, such as the opioid receptor or the cannabinoid receptor [22,23]. In addition, probiotics have been shown to reduce intestinal cytokine secretion and improve epithelial barrier function in a mice model of intestinal inflammation [24], and reduction of IBS symptoms in response to probiotic supplementation in subjects with IBS have been associated with improved cytokine profile [25].

Identification of specific bacterial strains or probiotic supplements with beneficial effects on IBS symptoms may lead to more effective therapy strategies. The theory that probiotic supplements improve IBS symptoms through modulation of the gut microbiota or its metabolic pathways needs further mechanistic evidence [26]. Hence, the aim of this systematic review was to assess the most recent randomized controlled trials (RCTs) evaluating the effect of probiotic supplementation on symptoms in IBS patients.

## 2. Materials and Methods

The checklist and flowchart of the PRISMA (Preferred Reporting for Systematic Reviews and Meta-Analyses) guidelines were followed for this systematic review [27].

### Search Strategy and Criteria for Inclusion

A literature search was conducted until March 2019 in the database PubMed (MEDLINE). The MeSH terms used in the search were “Probiotic AND Irritable Bowel Syndrome”, and the search filters “Last five years”, “Randomized Controlled Trial”, and “Humans” were used.

The search identified 35 studies, of which 11 were eligible for inclusion. The 11 studies included for further review were all randomized, double-blinded, placebo-controlled studies looking at IBS-patients diagnosed according to the Rome III criteria and published the last five years. Inclusion and exclusion criterion are shown in Table 1. All trials evaluated IBS symptoms and if, or how, they were altered in IBS patients given probiotics or placebo.

## 3. Results

### 3.1. Included Studies

**Figure 1:**
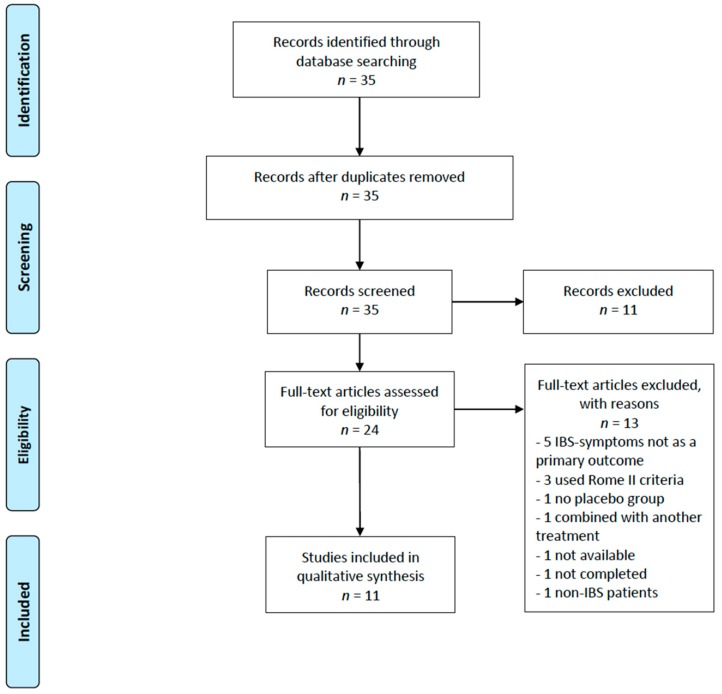
PRISMA flow diagram depicting the literature search in PubMed (Medline) for this systematic literature.

The search gave in total 35 studies, of which 11 articles were excluded based on their title. The studies excluded based on the title were either conducted in children, in non-IBS patients, or in healthy individuals, or they were studies evaluating the effect of a combination therapy on IBS symptoms. Within the 24 remaining studies, 13 studies were excluded based on the abstract or full-text review. Studies were excluded as symptoms were not reported as the primary outcome, probiotics were used in combination with other treatment, the trial did not include a control group, or the diagnosis of IBS was not done according to the Rome III criteria. In total, 11 studies evaluating the effect of probiotic supplementation on IBS symptoms were included in the systematic review. A flow diagram of the search process is depicted in Figure 1. All studies had a randomized, double-blinded, placebo-controlled trial methodology and included IBS patients diagnosed according to the Rome III criteria. The studies were conducted in either Europe or Asia. A summary of the included studies is listed in Table 2.

### 3.2. Main Findings

The main findings are summarized in Table 3. Among the eleven studies included in this review, the reported main findings are overall inconsistent. Seven out of the eleven studies (63.6%) reported that supplementation with probiotics in IBS patients significantly improved symptoms compared to placebo [29,32,33,35,36,37,38]. The remaining four studies (36.4%) did not report any significant improvement by probiotic supplementation; three (27.3%) reported small, but not significant, improvements [28,30,34], and one (9%) reported a suggested negative effect of probiotic supplementation compared to placebo [31].

Notably, of the eleven studies included, three studies evaluated the effect of a mono-strain supplement (containing only one strain of microorganism) [28,29,30], whereas the remaining eight trials supplemented with different combinations of two to fourteen different microorganisms (multi-strain) [31,32,33,34,35,36,37,38]. One of the three studies (33,3%) administering a mono-strain probiotic reported a significant improvement in IBS-symptoms [29], whereas six of the eight studies (75%) administering a multi-strain probiotic reported a significant improvement in IBS-symptoms [32,33,35,36,37,38]. The multi-strain probiotics used in the studies are all different from each other with regard to strain combination; however, they all use bacterial strains, and some strains and strain combinations are more prevalent than others. *Bifidobacterium* and *Lactobacillus* are the two bacterial genera most prevalent among the probiotics administered in the included studies, in line with the vast majority of probiotics on the commercial marked.

The supplementation practice for the multi-strain probiotics differed between studies both in quantity and frequency. Four of the studies investigating a multi-strain supplement had supplementation with probiotic capsules twice a day [31,32,33,38], whereas the rest of the studies only implemented one supplementation a day. Two of the four studies (50%) supplementing twice a day reported beneficial effects of the supplementation. Notably, the doses of probiotics in each supplementation varied greatly between studies and are not comparable (see amount reported in Table 2). Hence, the effect of frequency of supplementation may arguably be of less relevance than the amount of probiotics in each supplement/capsule, and the effects of frequency of supplementation cannot be concluded based on current data.

The timeframe of the different studies varied from 4 to 16 weeks of intervention, and the size of the study populations varied between 36 and 400 individuals. Additionally, the tools used for evaluation of symptom severity varied among the studies, where some used a physician’s evaluation and others used evaluation tools such as irritable bowel syndrome symptom severity score (IBS-SSS), visual analog scale (VAS), and standardized bowel disease questionnaire (SBDQ). The criteria for exclusion of participants also varied between studies. Some studies excluded participants with lactose intolerance, use of medication and/or other IBS treatment, whereas other studies did not. The studies that did not exclude participants that underwent other forms of treatment had specific criteria for how long the other treatment should have lasted before starting their intervention, and specified that the treatment should not be changed during the study.

### 3.3. Studies Evaluating the Effect of Mono-Strain Probiotics

Three of the included studies evaluated the effect of a mono-strain probiotic on IBS symptoms [28,29,30] (Table 3). None of these studies used the same strain of microorganism in their probiotic supplement. Two studies administered a supplement containing a bacterial strain (*Bacillus coagulans* and *Lactobacillus acidophilus*, respectively) [28,29] and one administered a supplement containing a yeast (*Saccharomyces cerevisiae*) [30]. Majeed et al. reported a significant effect of a mono-strain probiotic in improving IBS symptoms [29], whereas the remaining two did not report any significant effect of mono-strain probiotics in improving IBS symptoms [28,30].

Majeed et al. conducted a study including 36 irritable bowel syndrome with predominantly diarrhea (IBS-D) patients, with 90 days of probiotic supplementation [29]. The probiotic supplement consisted of the bacteria *Bacillus coagulans* MTCC5856 in two tablets administered once daily. After intervention, they reported a significant improvement in the intervention group compared to placebo in all primary outcomes: Bloating (*p* = 0.0037), vomiting (*p* = 0.0013), diarrhea (*p* = 0.0026), abdominal pain (10-point VAS score reduction from baseline: −3.89 ± 0.11, *p* = 0.0001), stool frequency (*p* = 0.0031), and consistency (*p* = 0.001). Additionally, they reported a significant improvement in the intervention group in secondary outcomes (a physician’s evaluation of global disease severity and QoL scores) compared to placebo group [29].

Lyra et al. conducted a trial including 391 participants with IBS [26]. They studied a low-dose and a high-dose probiotic supplement containing 10^9^ and 10^10^ CFU of *Lactobacillus acidophilus* NCFM, respectively. The probiotic was administered once daily over a period of 12 weeks. The participants were split into three groups: Placebo, low-dose and high-dose. IBS symptoms improved in all three groups with a mean IBS-SSS sum score reduction from baseline corresponding to −44.0 ± 80.2, −50.8 ± 82.4, and −48.3 ± 72.2, respectively. However, there was not a statistically significant difference between the groups [28].

Pineton et al. studied the effect of the yeast *Saccharomyces cerevisiae* CNCM I-3856 administered as a probiotic supplement in a capsule taken once daily [28]. The study was an 8-week intervention with a 2-week follow-up, and included 179 adults with IBS. The primary outcome was change in abdominal pain/discomfort. Both the intervention group and the placebo group had significant improvement from baseline to after intervention, with a reduction in abdominal pain score (7-point scale) of 37.2% and 26.9%, respectively. However, there was no statistically significant difference between the placebo and intervention group (*p* = 0.13) [30].

### 3.4. Studies Evaluating the Effect of Multi-Strain Probiotics

Eight studies evaluated the effect of multi-strain probiotics in IBS patients [31,32,33,34,35,36,37,38] (Table 3). The multi-strain probiotics administered in the eight studies were all different from each other, but some strains and strain combinations were more prevalent than others. Seven of the studies administered multi-strain probiotics that were sold as nutritional supplements with a commercial name, branding, and copyright [31,32,33,34,36,37,38], whereas Mezzasalma et al. administered two different supplements produced for research purposes only [35].

Among the eight studies evaluating the effect of a multi-strain probiotic supplement, two studies did not report any beneficial effects [31,34]; Ludidi et al. conducted a study in 40 patients hypersensitive to rectal distention [34]. The probiotic supplement contained six different bacterial strains and it was administered as a powder mixed in cold water, taken once daily for 6 weeks. The primary outcome was change in visceral perception. The percentage of patients with visceral hypersensitivity decreased in both the probiotic and the placebo group (76.5% and 71.4%, respectively); however, the response did not differ between the groups (*p* = 0.25) [34].

Similar results were reported in a study by Hod et al. investigating the effect of a probiotic capsule given twice daily for 8 weeks in 107 females with IBS-D [31]. The capsule contained 12 different bacterial strains (listed in Table 2). Study outcomes included abdominal pain, bloating, and urgency and frequency of bowel movements. All symptoms improved significantly in both groups, but no differences were observed between placebo and probiotics. Abdominal pain was reduced with a median 10-point VAS score from baseline of 0.97 in the probiotic group and 1.79 in the placebo group (*p* = 0.203) [31].

Six of the eight studies evaluating the effect of a supplement with multi-strain probiotics found a significant improvement in IBS symptoms; three of these studies found a significant improvement in abdominal pain using a multi-strain probiotic supplement [32,33,38]. Ishaque et al. conducted a study in 400 participants with an intervention period of 16 weeks [32]. The study included patients with subtype IBS-D with moderate to severe IBS symptoms. Their probiotic supplement consisted of 14 different bacterial strains and was given as two capsules taken twice daily. Symptoms were evaluated by IBS-SSS, and the probiotic treatment was found to significantly improve abdominal pain in the intervention group (69% improvement from baseline) compared to the placebo group (47% improvement from baseline) (*p* < 0.001). The secondary outcomes—improvements in general IBS symptoms and QoL,—were also reported to be significantly higher in the intervention group [32].

Improvement in abdominal pain was also reported in a study by Jafari et al. that evaluated the effect of a probiotic supplement containing *Bifidobacterium animalis*, *Lactobacillus acidophilus*, *L. delbrueckii bulgaricus,* and *Streptococcus thermophilus* [33]. The study included 108 participants and had a 4-week intervention period. Outcomes included change in abdominal pain and bloating, evaluated by a 100 mm VAS score. Both outcomes improved significantly in the probiotic group compared to placebo, with a reduction in abdominal pain of −8.2 vs. −2.1 (*p* = 0.02) and abdominal bloating of −13.0 vs. −3.7 (*p* ≤ 0.01) from baseline to after intervention, respectively. After subgroup analysis, no significant difference between genders was reported [33].

Wong et al. also reported improvement in abdominal pain after supplementation with a probiotic supplement containing eight different bacterial strains administered as a capsule taken 4 × 2 daily for 6 weeks [38]. The study included 42 adults with IBS. Primary outcome was a change in IBS-SSS score (100 mm VAS score). They reported significant improvement in pain duration (−18.5 vs. −7.28, *p* ≤ 0.05) and abdominal distention (−14.50 vs. 12.28, *p* ≤ 0.05) in the intervention group compared to the placebo group, respectively. When patients were sub-grouped by gender, a statistically significant difference was found between intervention group males and placebo group males in IBS severity, but this difference was not found between females in the two groups [38].

The three remaining studies all reported significant improvement in general symptom relief in IBS patients [35,36,37]. Mezzasalma et al. conducted a study including 157 participants diagnosed with irritable bowel syndrome with predominately constipation (IBS-C), evaluating the effect of a supplement containing two different types of multi-species probiotics in two different intervention groups (F1 and F2), compared to a placebo group [35]. The F1 probiotic consisted of *Lactobacillus acidophilus* and *L. reuteri*, and the F2 probiotic consisted of *L. planetarium, L. rhamnosus,* and *Bifidobacterium animalis lactis*. Symptom outcomes included bloating, abdominal pain, constipation, abdominal cramps, and flatulence. A significant improvement was reported overall in both intervention groups (F1 and F2) compared to placebo (F3), with the percentage of responders for each clinical symptom being significantly higher in both of the probiotic groups compared to the placebo group (66–90% vs. 6–36%, *p* < 0.001) [35].

Similar results were found in a study by Sisson et al. that evaluated the effect of a liquid probiotic containing *Lactobacillus rhamnosus*, *L. planetarium*, *L. acidophilus,* and *Enterococcus faecium* [36]. The probiotic was administered once daily for 12 weeks in 186 IBS patients. The primary outcome was overall improvement in IBS-SSS scores, and secondary outcomes were change in QoL scores and change in the different IBS-SSS sub-scores after intervention and after a 4-week follow-up. They reported a significant improvement in overall IBS symptoms in the probiotic group compared to placebo, with a reduction of total IBS-SSS score of −63.3 ± 87.9 and −28.3 ± 81.2 from baseline to after intervention, respectively (*p* = 0.01). Non-significant differences between the two groups were reported at the 4- and 8-week check-ups [36].

These beneficial findings are supported by a study by Staudacher et al. evaluating the effect of both probiotic supplementation and low-FODMAP diet on IBS symptoms [37]. The study included 104 participants allocated to four groups: One placebo and sham diet group, one placebo and low-FODMAP group, one probiotic and sham diet group, and one probiotic and low-FODMAP group. Only the results from the placebo and sham diet group (*n* = 27) and probiotic and sham diet group (*n* = 26) were included in this review. The probiotic supplement contained eight different bacterial strains (see Table 2). The probiotic group had a significantly higher proportion of reported “symptom relief” than the placebo group, with a symptom reduction of 57% vs. 37% from baseline to after 4 weeks of intervention, respectively (*p* = 0.048) [37].

### 3.5. Beneficial Probiotic Species

Although none of the included trials used the same probiotic mixture, some tendencies regarding efficiency of different species were observed. In the six studies reporting beneficial effects of multi-strain probiotics, several species were represented in the majority of the studies. The most frequent was *Lactobacillus acidophilus*, represented in all supplements used in the multi-strain studies reporting beneficial effects [32,33,35,36,37,38]. *Streptococcus thermophilus* was present in four of the multi-strain supplements reported to have beneficial effects [32,33,37,38], whereas *Bifidobacterium breve* and *Bifidobacterium longum* in combination were used in three of the multi-strain supplements found to be efficient [32,37,38]. The current data do not enable evaluation of trends regarding specific strains of each species.

## 4. Discussion

Overall, the studies included in the current review report varying results with regard to the effect of a probiotic supplement on IBS symptoms. When comparing studies administering multi-strain probiotic supplements with studies administering mono-strain probiotic supplements, the tendency is a more beneficial effect of multi-strain probiotic treatment compared to placebo and mono-strain probiotic treatment in alleviating IBS symptoms. Of note, the small sample size does not enable a conclusion, but rather suggests a trend.

Our findings are consistent with several recent meta-analyses and systematic reviews highlighting that probiotics in general have significant, however limited, effects on gastrointestinal symptoms [39,40,41]. A review and meta-analysis by Ford et al., including RCTs published between 1946 and 2013 evaluating probiotics as treatment for IBS symptoms, concluded that probiotics had a beneficial effect on IBS symptoms, and emphasized that the effect was more distinct when using multi-strain probiotics [40]. Results from another more recent review and meta-analysis by Ford et al., evaluating the efficacy of probiotics, prebiotics, and antibiotics in IBS, support their previous publication. They concluded that particular combinations of probiotics, or specific species and strains, appeared to have beneficial effects on general IBS symptoms and abdominal pain; however, based on current evidence, it was not possible to conclude in detail on which particular combination is the most efficient [39]. Hungin et al. recently performed a systematic review reporting on beneficial effects of specific probiotic supplements on lower gastrointestinal problems in IBS, and highlighted great differences observed between different types and strains of probiotic supplements [41].

The type of probiotic supplement administered in the trials included in this review are all different from each other according to form, amount, microbial strains, and combinations of microbial strains (see Table 2). The separation between studies that administered mono- and multi-strain probiotic supplements presented an important difference in results across these two groups of studies, which is consistent with results from the meta-analysis by Ford et al. [40]. Furthermore, there is a wide variety between the multi-strain probiotic supplements administered in the included studies. However, the two most common bacterial families administered as probiotics in the included studies were the *Lactobacillaceae* and *Bifidobacteriaceae* (genus: *Lactobacillus* and *Bifidobacterium*), and all of the eight studies that administered a multi-strain probiotic supplement in their study had either one or both of these two bacterial families included in their supplement. In former studies conducted to examine the fecal microbiota of IBS patients, counts of Lactobacillus were reported to be both heightened and lowered in IBS patients compared to healthy controls in different studies [11,42], and more studies are still needed to affirm any tendencies in *Lactobacillus* counts in IBS patients. Bifidobacterium, on the other hand, has been reported by former studies to be found exclusively in reduced amounts in fecal samples of IBS patients [12,16,43], which supports the findings in the current study, indicating that it is a tendency of significant improvement in symptoms of IBS patients consuming a multi-strain probiotic containing this bacterial family. Still, what strains and what combination of strains that are most effective remains unclear and needs further investigations.

The duration of intervention varied between the eleven studies and spanned from 4 to 16 weeks. Sisson et al. had an intervention period of 12 weeks and reported a significant improvement in the intervention group compared to the placebo group after finished intervention [36]. However, they did not find a significant difference between groups at the 4-week and 8-week check-ups. This proposes a potential delayed effect of probiotic supplementation in reducing IBS symptoms, which may give a non-significant result in shorter studies (≤8 weeks). Three of the four studies (75%) reporting a non-significant improvement in IBS symptoms had an intervention period of 8 weeks or less [31,33,34], whereas four out of seven of the studies (57.2%) reporting significant improvements in symptoms had an intervention period of 8 weeks or more. These findings suggest that probiotic supplements have a delayed effect in the improvement of IBS symptoms.

Several issues have to be considered when interpreting the present results. Firstly, the current review only includes RCTs published in the last five years, thus does not include relevant findings from earlier publications. Secondly, methodological differences between studies, such as type of probiotic, duration of intervention, sample size, and symptom evaluation, might affect the findings. In addition, the included studies used different symptom evaluation tools. Among the eleven included studies, the validated IBS-SSS [44] was the most frequently used questionnaire for symptom evaluation, implemented in five of the trials [28,32,36,37,38]. Based on the validity of IBS-SSS, we suggest this for standardized use in future studies.

The treatment of IBS should ideally be based on IBS subtype. However, there are limited results to support such a practice, and multiple treatments may be tested before the patient experiences symptom relief. Still, there is some evidence of subtype-specific treatments such as the low-FODMAP diet, which gives the best results in patients with IBS-D [45], and supplementation with psyllium husk which gives best results in IBS-C [46]. In the current review, one study included all subtypes of IBS and conducted a separate analysis to evaluate any potential differences in symptom improvement in the different IBS subtypes; however, no significant differences were found between subgroups [30]. Four studies included participants with only one subtypes of IBS; three studies only included participants with IBS-D [29,31,32]; and one study only included participants with IBS-C [35]. Two of the studies including participants with IBS-D reported significant improvement in symptoms after probiotic supplementation [29,32], whereas one study reported no significant improvement in symptoms and even reported a suggested negative effect of probiotic supplementation compared to placebo [31]. The one study only including IBS-C patients reported a significant improvement in increased number of symptom-free days and quality of life [35]. Future studies should aim to evaluate the effect of probiotic supplementation comparing different subtypes of IBS, which requires large sample sizes where all IBS subtypes are sufficiently represented.

IBS has a higher prevalence in women than men [1], and a trial by Camilleri et al. reported gender differences in IBS patients in response to a pharmaceutical treatment (Alosetron, a 5-HT3receptor antagonist) [47]. Three of the included studies conducted a separate analysis to evaluate gender differences in their study population [33,34,38]. Wong et al. reported a difference in symptom improvement between gender, emphasizing that the male intervention group had a significant improvement in IBS symptoms compared to males of the placebo group, whereas the female participants in the intervention group did not show the same significant improvement compared to females in the placebo group [38]. None of the two remaining studies reported any significant difference between gender [33,34]. Notably, Hod et al. included only females in the trial [31]. Based on the reported results in the current review, there seem to be no difference between male and female participants in symptom improvement after consuming a probiotic supplement. However, there is still not enough data on how potential gender differences may interfere with probiotic treatment of IBS, and large studies with separate gender analyses are needed in the future to declare any potential differences between male and female IBS patients.

Overall, the concept of the human microbiome and the dysbiotic gut as a target for novel therapeutic strategies for improving gastrointestinal symptoms in IBS suggests that more individualized and tailored probiotic supplements will be available in the future. In this context, *Akkermansia*, *Bacteroides,* and *Faecalibacterium* are the microbes proposed to be relevant for further investigation [48].

## 5. Conclusions

When comparing the results from studies administering a multi-strain versus a mono-strain probiotic supplement, the overall observed tendency is that a supplement with a multi-strain probiotic has the potential to improve IBS symptoms. The specific symptoms improved by probiotic supplementation were not consistent between studies. Some studies found a general improvement in IBS symptoms, whereas others reported improvement in specific symptoms like abdominal pain and bloating. The studies using a mono-strain probiotic supplement did not report the same affirmative tendency for probiotic supplementation as the studies using a multi-strain supplement. In studies that conducted separate subgroup analysis, there was no clear tendency in differences between genders or IBS subtype on the effect of probiotic treatment.

Based on the current findings, future studies should aim to further evaluate the effect of multi-strain probiotic supplementation on IBS symptoms in RCTs with an intervention period lasting more than 8 weeks. Standardized symptom questionnaires, preferably IBS-SSS, should be implemented to acquire more knowledge about what strains are the most effective, and which subtype of IBS patients that potentially benefit the most from probiotic treatment.

## Figures and Tables

**Table 1:** Inclusion and exclusion criteria for the systematic literature review.

Inclusion	Exclusion
IBS patients	Healthy adults or non-IBS patients
Human studies	Animal studies
Studies in adults (over 18 years)	Studies in children
RCT studies	Studies without RCT methodology
Double- or triple-blinded studies	Single-blinded or partially blinded studies
Studies published the last five years	Studies older than five years
IBS diagnosis with Rome III or Rome IV criteria	IBS diagnosis with Rome II or Manning criteria
Studies looking at change in IBS symptoms as primary outcome	Studies not looking at change in IBS symptoms as primary outcome
Studies looking solemnly at probiotics in an intervention group	Studies looking at probiotics in conjunction with other IBS therapy in the same intervention group

IBS, irritable bowel syndrome; RCT, randomized controlled trial.

**Table 2:** Overview of the 11 studies included in the systematic review.

First Author, Year of Publication, Country	*N*	Probiotic Strains (Amount)	Dose	Probiotic Form	IBS Subtype	Gender	Study Duration	Symptom Evaluation
Mono-strain probiotics								
Lyra, 2016, Finland [28]	391	*Lactobacillus acidophilus* NCFM (low dose: 10^9^ CFU, high dose: 10^10^ CFU)	Once daily	Capsule	Not specified	Both	12 weeks	IBS-SSS, QoL, and HADS
Majeed, 2016, India [29]	36	*Bacillus coagulans* MTCC5856 (2 × 10^9^ CFU)	Once daily	Tablet	IBS-D	Both	90 days	VAS, Physician’s evaluation, and QoL
Pineton, 2015, France [30]	179	*Saccharomyces cerevisiae* CNCM I-3856 (500 mg, 8 × 10^9^ CFU/g)	Once daily	Capsule	Not specified	Both	8 weeks	7-point Likert scale and CMH
Multi-strain probiotics								
Hod, 2017, Israel [31]	107	*Lactobacillus rhamnosus* LR5 (3 × 10^9^ CFU), *L. casei* LC5 (2 × 10^9^ CFU), *L. paracasei* LPC5 (1 × 10^9^ CFU), *L. plantarum* LP3 (1 × 10^9^ CFU), *L. acidophilus* LA1 (5 × 10^9^ CFU), *Bifidobacterium bifidum* BF3 (4 × 10^9^ CFU), *B. longum* BG7 (1 × 10^9^ CFU), *B. breve* BR3 (2 × 10^9^ CFU), *B. infantis* BT1 (1 × 10^9^ CFU), *Streptococcus thermophilus* ST3 (2 × 10^9^ CFU), *L. bulgaricus* LG1, and *Lactococcus lactis* SL6 (3 × 10^9^ CFU)	Twice daily	Capsule	IBS-D	Female	8 weeks	VAS
Ishaque, 2018, Bangladesh [32]	400	*Bacillus subtilis* PXN 21, *Bifidobacterium bifidum* PXN 23, *B. breve* PXN 25, *B. infantis* PXN 27, *B. longum* PXN 30, *Lactobacillus acidophilus* PXN 35, *L. delbrueckii. Bulgaricus* PXN39, *L. casei* PXN 37, *L. plantarum* PXN 47, *L. rhamnosus* PXN 54, *L. helveticus* PXN 45, *L. salivarius* PXN 57, *Lactococcus lactis* PXN 63, and *Streptococcus thermophilus* PXN 66 (in total 8 × 10^9^ CFU)	2 × twice daily	Capsule	IBS-D	Both	16 weeks	IBS-SSS
Jafari, 2014, Iran [33]	108	*Bifidobacterium animalis lactis* BB-12, *Lactobacillus acidophilus*, *L. delbrueckii bulgaricus* LBY-27, and *Streptococcus thermophilus* STY-31 (in total approx. 4 × 10^9^ CFU)	Twice daily	Capsule	Not specified	Both	4 weeks	VAS
Ludidi, 2014, The Netherlands [34]	40	*Bifidobacterium lactis* W52, *Lactobacillus casei* W56, *L. salivarius* W 57, *L. acidophilus* NCFM, *L. rhamnosus* W71, and *Lactococcus lactis* W58 (in total 5 × 10^9^ CFU)	Once daily	Powder mixed in water	Not specified	Both	6 weeks	5-point Likert scale, barostat, and VAS
Mezzasalma, 2016, Italy [35]	157	F1: *Lactobacillus acidophilus* (5 × 10^9^ CFU) and *L. reuteri* (5 × 10^9^ CFU); F2: *Lactobacillus plantarum* (5 × 10^9^ CFU), *L. rhamnosus* (5 × 10^9^ CFU), and *Bifidobacterium animalis lactis* (5 × 10^9^ CFU)	Once daily	Capsule	IBS-C	Both	60 days	VAS and QoL
Sisson, 2014, United Kingdom [36]	186	*Lactobacillus rhamnosus, L. plantarum, L. acidophilus,* and *Enterococcus faecium* (in total approx. 10^10^ CFU)	Once daily	Liquid	Not specified	Both	12 weeks	IBS-SSS
Staudacher, 2017, United Kingdom [37]	53	*Streptococcus thermophilus* DSM 24731, *Bifidobacterium breve* DSM 24732, *B. longum* DSM 24736, *B. infantis* DSM 24737, *Lactobacillus acidophilus* DSM 24735, *L. plantarum* DSM 24730, *L. paracasei* DSM 24733, and *L. delbrueckii. bulgaricus* DSM 24734 (in total 11,95 log_10_ CFU)	Once daily	Powder mixed in water	IBS-D, -M, and -U	Both	4 weeks	IBS-SSS
Wong, 2015, Singapore [38]	42	*Bifidobacterium longum, B. infantis, B. breve, Lactobacillus acidophilus, L. casei, L. delbrueckii bulgaricus, L. plantarum,* and *Streptococcus salivarius thermophilus* (in total 112.5 billion)	4 × 2 daily	Capsule	Not specified	Both	6 weeks	IBS-SSS, HADS, SBDQ, and barostat

CFU, colony forming units; CMH, Cochran–Mantel–Haenszel test; HADS, hospital anxiety and depression scale; IBS-C, irritable bowel syndrome with predominately constipation; IBS-D, irritable bowel syndrome with predominantly diarrhea; IBS-M, irritable bowel syndrome with a mixture of both diarrhea and constipation; IBS-U, irritable bowel syndrome uncategorized; IBS-SSS, irritable bowel syndrome symptom severity score; *N*, sample size; QoL, quality of life, SBDQ, standardized bowel disease questionnaire; VAS, visual analog scale.

**Table 3:** Overview of outcome measures and main findings in the eleven trials included in this systematic review.

First Author, Year Published, Country	Primary Outcome	Main Findings, Primary Outcome	Secondary Outcome	Main Findings, Secondary Outcome
Mono-strain probiotics				
Lyra, 2016, Finland [28]	Change in IBS-SSS scores	Sign. improvement in all three groups, but no sign. difference between groups	Change in anxiety and depression scores and adequate relief	No sign. differences between groups
Majeed, 2016, India [29]	Bloating, vomiting, diarrhea, abdominal pain, stool frequency and consistency	Sign. improvement in all mentioned symptoms in IG compared to PG	Physician’s global assessment of disease severity and QoL	Sign. difference in improvement of disease severity and QoL
Pineton, 2014, France [30]	Change in abdominal pain/discomfort	Sign. improvement in both group from baseline, but no sign. difference between IG and PG	Change in bloating/distention and bowel movement difficulty	Sign. improvement in both group from baseline, but no sign. difference between them
Multi-strain probiotics				
Hod, 2017, Israel [31]	Degree of symptom relief	Sign. improvement in both groups. Only sign. result was an improvement in abdominal pain in the PG compared to IG	Bloating, urgency, and frequency of bowel movements and inflammatory markers (FC and Hs-CRP)	No sign. results
Ishaque, 2018, Bangladesh [32]	The change in severity and frequency of abdominal pain on the IBS-SSS	Abdominal pain level decreased by 40 points in the IG versus a 27 point decrease in the PG	The change in other GI symptom severity scores on the IBS-SSS, QoL, and AEs	Sign. improvement in IG in IBS symptoms and QoL
Jafari, 2014, Iran [33]	Reduction in mean abdominal bloating score and “satisfactory relief”	The IG had an improvement in abdominal bloating. Satisfactory relief was 85% reduced in IG compared to 47% in PG	Changes in abdominal pain or discomfort score and reduction in feeling of incomplete defecation	Feeling of incomplete defecation was reduced in IG abdominal pain scores were lower in IG compared to PG after one month follow-up
Ludidi, 2014, The Netherlands [34]	Visceral perception	Sign. improvement in both groups, but no difference between them	Symptom scores (number of symptom-free days and MMS)	PG showed a decrease in MMS, but the difference between the groups was not significant. No sign. difference in number of symptom-free days
Mezzasalma, 2016, Italy [35]	Number of symptom-free days and fecal microbiota	Sign. improvement in the two IGs compared to PG in number of symptom-free days and increased amount of the supplemented species in the two IGs feces samples	Maintenance of effect after 30 d of wash-out and QoL	Maintenance of intervention effect was sign. higher in the two IGs than in the PG improvement in QoL in IGs compared to PG
Sisson, 2013, United Kingdom [36]	Change in overall IBS-SSS scores	Sign. improvement in overall symptoms in IG compared to PG	QoL and change in IBS-SSS components after 12 w intervention and 4 w follow-up	Improvement in bowel movements was sign. better in the IG. No difference in QoL scores. No sign. results after 4 w
Staudacher, 2017, United Kingdom [37]	“Adequate symptom relief” and fecal *Bifidobacterium* species concentration	Adequate symptom relief was reported in 57% in IG compared to 37% in PG. A greater abundance of the *Bifidobacterium* was found in the IG compared to PG	Individual GI symptoms (IBS-SSS and GSRS), stool output, HRQOL, microbiota diversity, and nutrient intake	Flatulence scores were sign. lower in IG compared to PG. No other sign. differences were found between the groups
Wong, 2015, Singapore [38]	Change in IBS-SSS scores	Change in pain duration and abdominal distention decreased sign. in IG compared to PG	Rectal sensitivity and saliva melatonin levels	No sign. results

AEs, adverse events; d, days; FC, fecal calprotectin; GI, gastrointestinal; GSRS, gastrointestinal symptom rating scale; HRQOL, health-related quality of life; Hs-CRP, high sensitive C-reactive protein; IG, intervention group; IBS, irritable bowel syndrome; IBS-SSS, irritable bowel syndrome symptom severity score; IL, interleukin; MMS, mean symptom composite score; PG, placebo group; QoL, quality of life; Sign, significant; TNF, tumor necrosis factor; w, week.
